# Fisetin Lowers Methylglyoxal Dependent Protein Glycation and Limits the Complications of Diabetes

**DOI:** 10.1371/journal.pone.0021226

**Published:** 2011-06-27

**Authors:** Pamela Maher, Richard Dargusch, Jennifer L. Ehren, Shinichi Okada, Kumar Sharma, David Schubert

**Affiliations:** 1 Cellular Neurobiology Laboratory, The Salk Institute for Biological Studies, La Jolla, California, United States of America; 2 Department of Medicine, Center for Renal Translational Medicine, University of California San Diego, La Jolla, California, United States of America; Biological Research Center of the Hungarian Academy of Sciences, Hungary

## Abstract

The elevated glycation of macromolecules by the reactive dicarbonyl and α-oxoaldehyde methylglyoxal (MG) has been associated with diabetes and its complications. We have identified a rare flavone, fisetin, which increases the level and activity of glyoxalase 1, the enzyme required for the removal of MG, as well as the synthesis of its essential co-factor, glutathione. It is shown that fisetin reduces two major complications of diabetes in Akita mice, a model of type 1 diabetes. Although fisetin had no effect on the elevation of blood sugar, it reduced kidney hypertrophy and albuminuria and maintained normal levels of locomotion in the open field test. This correlated with a reduction in proteins glycated by MG in the blood, kidney and brain of fisetin-treated animals along with an increase in glyoxalase 1 enzyme activity and an elevation in the expression of the rate-limiting enzyme for the synthesis of glutathione, a co-factor for glyoxalase 1. The expression of the receptor for advanced glycation end products (RAGE), serum amyloid A and serum C-reactive protein, markers of protein oxidation, glycation and inflammation, were also increased in diabetic Akita mice and reduced by fisetin. It is concluded that fisetin lowers the elevation of MG-protein glycation that is associated with diabetes and ameliorates multiple complications of the disease. Therefore, fisetin or a synthetic derivative may have potential therapeutic use for the treatment of diabetic complications.

## Introduction

The complications of diabetes are the major cause of both morbidity and mortality in patients with the disease [1], [2]. Chronic hyperglycemia is thought to be a major cause of these complications and the downstream consequences of hyperglycemia include multiple pathophysiological processes including protein glycation, reactive oxygen species (ROS) production and inflammation [3], [4]. We have recently identified and characterized the flavonoid fisetin as an orally active neuroprotective and cognition-enhancing molecule [5]. Fisetin protects nerve cells in culture from assorted toxic insults including ischemia and oxidative stress. It has direct antioxidant activity and also increases the level of the major intracellular antioxidant, glutathione (GSH). In the context of diabetic complications, GSH is an essential co-factor for glyoxalase 1 (Glo-1), the rate-limiting enzyme in the removal of methylglyoxal (MG). Fisetin can also induce the transcription factor Nrf2 that is associated with the up-regulation of GSH metabolism and the protection of cells from toxic stress. Others have found that fisetin has anti-inflammatory activity *in vitro* [6], [7]. Although many of these activities of fisetin have the potential to reduce the metabolic dysfunctions that are associated with diabetic complications, it is not clear which activities are relevant *in vivo*.

Protein glycation, especially by MG, may be a central player in the complications of diabetes because it has the ability to increase both inflammation and oxidative stress [8], [9]. Glycation is the result of the non-enzymatic addition of sugars and toxic aldehydes such as MG and glyoxal to macromolecules and results in the formation of advanced glycation end-products (AGEs). Of the potential glycating agents, MG is the most effective, and its production is directly coupled to extracellular glucose levels and therefore the rate of glycolysis [10]. Protein glycation is greatly enhanced in diabetic tissues, and it has been suggested that glycation is a major cause of diabetic complications [11], [12].

To test the hypothesis that a compound such as fisetin which targets multiple pathways associated with hyperglycemia including MG-dependent protein glycation *in vitro* can prevent the complications of diabetes *in vivo*, we examined the effects of fisetin on the development of diabetic nephropathy and anxiety symptoms in the Akita mouse model of type 1 diabetes. Importantly, a recent epidemiological provided evidence that diabetic kidney disease is a marker of brain dysfunction in patients with type 1 diabetes [13]. We show that the treatment of Akita mice with fisetin not only dramatically reduces diabetic kidney damage but also lowers protein glycation by MG. The latter observation is consistent with the idea that protein glycation may be a major contributing factor to the complications of diabetes.

## Results

Fisetin is orally active and has a number of properties *in vitro*, including anti-oxidant and anti-inflammatory activities, which have the potential to mitigate multiple complications of diabetes. Akita mice have a mutation in the insulin gene and develop the pathological characteristics of type 1 diabetes, including diabetic nephropathy [14] and elevated anxiety symptoms [15], [16]. Therefore, it was asked whether fisetin could reduce these complications. Four groups of mice were studied, 2 groups each of wild type and Akita. Fisetin was fed to one set of control and one set of Akita mice between the ages of 6 and 24 weeks in their food at 0.05%, resulting in a daily dose of approximately 25–40 mg/kg. This dose of fisetin was chosen based on earlier studies on fisetin and cognitive function in mice [17]. Male Akita mice develop hyperglycemia by 4 weeks of age [18]. We tested blood glucose in all animals at 12 weeks and again before sacrificing the animals at 24 weeks. At this time, blood glucose and HbA1c were significantly elevated in the Akita mice (Table 1). These changes were not affected by the presence of fisetin in the diet. The Akita mice also showed significant weight loss relative to their wild type counterparts and this was also not altered by fisetin in the diet. Fisetin had no effect on blood glucose and HbA1c levels in the wild type mice.

**Table 1:** Metabolic Parameters at the end of the study.

Phenotype	Weight (g)	Blood Glucose (mg/dl)	HbA_1c_ (%)
**Wild type**	29.5±2.2	52.2±32	4.9±0.2
**Wild type + fisetin**	32.8±3.1	73.0±29	5.2±0.4
**Akita**	23.9±1.7*	591.4±78*	17.7±3.5*
**Akita + fisetin**	23.0±2.3*	560.1±86*	16.0±1.6*

Statistical analysis by one-way ANOVA followed by Dunnett's post-hoc test to compare all groups against wild type. Data are mean ± SD of N = 5–7 per group.

* = P<0.001 vs. wild type.

### Fisetin reduces kidney damage in diabetic mice

**Figure 1:**
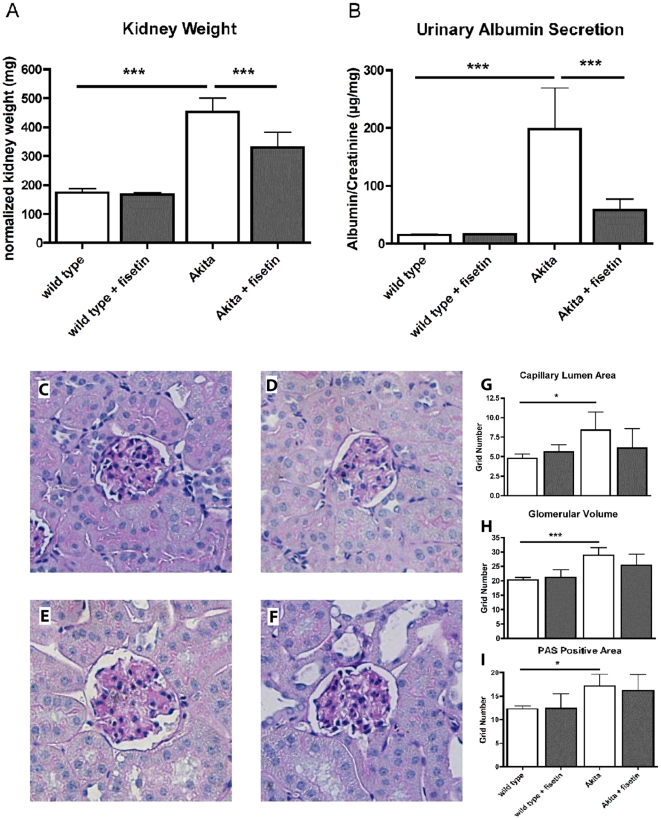
Fisetin reduces the consequences of diabetes in Akita mice. Male Akita mice were fed control diet or fisetin (0.05%) for 18 weeks beginning at 6 weeks of age. (A) Kidney hypertrophy was assessed by weighing the kidneys and normalizing the kidney weight to the relative weight of the mice. (B) Kidney function was assessed by measuring the albumin/creatinine ratio in the urine. Fisetin reduced diabetes-induced renal hypertrophy and albuminuria. Capillary lumen area, glomerular volume and PAS positive mesangial matrix area were increased in Akita mouse kidneys (E, F: +fisetin) compared with wild type mice (C, D: +fisetin). The increase in capillary lumen area (G) (*p*<0.05) and glomerular volume in the Akita mouse kidneys was significantly different from controls (H) (*p*<0.001) and was largely prevented by fisetin. The PAS positive area was also significantly increased in the Akita mouse kidneys (I) (*p*<0.05) while this difference was not significant in the Akita + fisetin kidneys. All data are mean ± SD of *n* = 5–7/group. *(*p*<0.05), **(*p*<0.01), ***(*p*<0.001) indicate significantly different from wild type or Akita alone as determined by ANOVA followed by Tukey's post test.

Kidneys of Akita mice were significantly heavier than those of wild type mice, consistent with the hypertrophy associated with diabetic nephropathy in this strain of mice [14]. The diabetes-induced increase in kidney size was significantly reduced by fisetin (Fig. 1A). Fisetin had no effect on kidney weight in wild type mice. In agreement with the increase in kidney weight, urine analysis revealed significant albuminuria in the Akita mice that was almost completely prevented by the presence of fisetin in the diet (Fig. 1B). Fisetin had no effect on the albumin/creatinine ratio in wild type mice.

Capillary lumen area, glomerular volume and PAS positive mesangial matrix area were all significantly increased in the kidneys of Akita mice as compared to wild type mice but not in the kidneys of fisetin-treated Akita mice (Fig. 1C–I).

### Fisetin protects diabetic mice from anxiety-associated behavior

**Figure 2:**
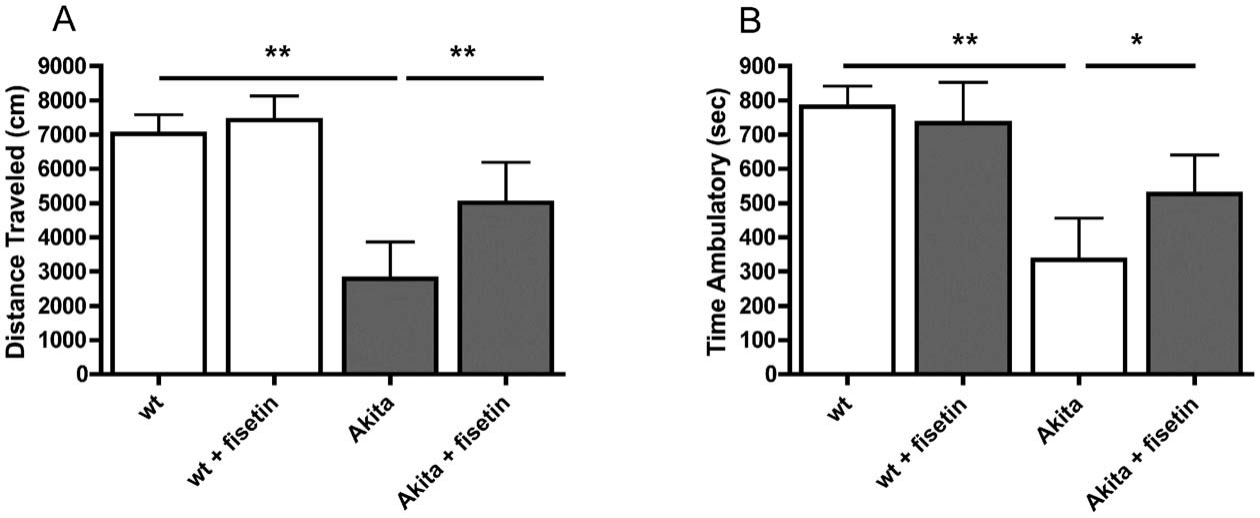
Fisetin reduces an experimental index of anxiety in Akita mice. Male Akita mice were fed control diet or fisetin (0.05%) diet for 18 weeks beginning at 6 weeks of age. Locomotor activity was measured in the open field test. Decreased locomotor activity is an indicator of elevated anxiety that is characteristic of diabetics in poor glycemic control. Fisetin restored (A) distance traveled and (B) time ambulatory to nearly control levels. All data are mean ± SD of *n* = 5–7/group. *(*p*<0.05) and **(*p*<0.01) indicate significantly different from wild type or Akita alone as determined by ANOVA followed by Tukey's post test.

CNS dysfunction is another complication of diabetes. Although cognitive impairment is associated with long-term diabetes in humans [19] and is seen in some mouse models of type 1 diabetes [20], [21], we saw no deficits in behavior in the Akita mice using the Morris water maze, an assay of spatial learning and memory (not shown), consistent with an earlier study [22]. However, Akita mice display significantly decreased locomotor activity in the open field test coupled with a significantly increased time of immobilization [15]. These results were interpreted as indicative of anxiety behavior, a CNS complication of many patients with diabetes [16]. As shown in Figure 2, we confirmed these results with the Akita mice. Furthermore, Akita mice fed fisetin showed a significant reduction in these anxiety-related symptoms with both the distance traveled and time ambulatory in the open field test restored to near normal values. Fisetin had no effect on the behavior of the wild type mice in the open field test.

### Fisetin reduces markers of oxidative stress

**Figure 3:**
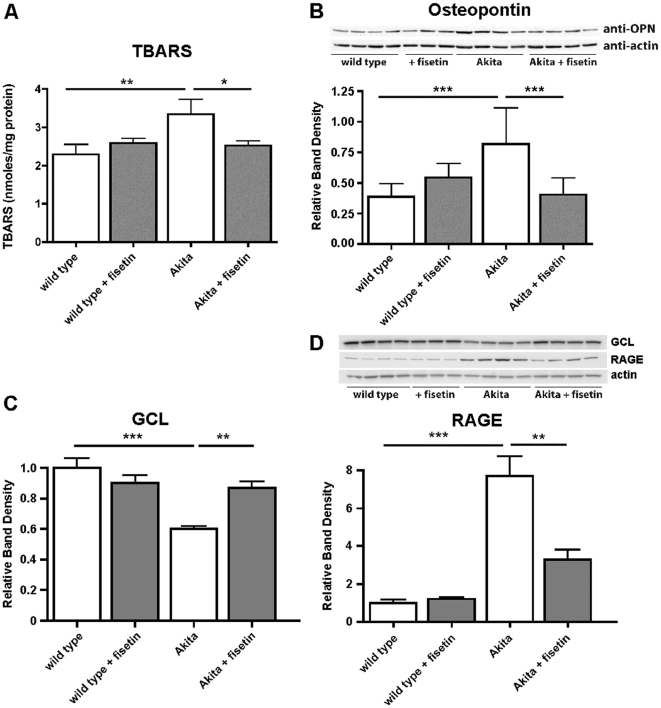
Fisetin reduces biochemical changes in Akita mouse kidneys. (A) The diabetes-dependent increase in TBARS is prevented by fisetin. Kidneys from wild type, wild type + fisetin, Akita and Akita + fisetin mice were homogenized in PBS containing phosphatase and protease inhibitors and equal amounts of extract were assayed for TBARS. Kidney samples from 6–7 mice were analyzed and the amount of TBARS normalized to protein. (B) The diabetes-dependent up-regulation of osteopontin (OPN) is prevented by fisetin. Kidneys from wild type, wild type+fisetin, Akita and Akita + fisetin mice were homogenized in PBS containing phosphatase and protease inhibitors and equal amounts of protein were analyzed by SDS-PAGE and Western blotting with an antibody to OPN and actin as a loading control. The expression of GCL (C) and the RAGE receptor (D) in kidneys were analyzed by Western blotting as described in (B). Kidney samples from 6–7 mice per group were analyzed and the average intensity of the GCL, RAGE, and OPN bands normalized to actin is shown in the graphs. A representative blot is shown for each. ***(*p*<0.001), **(*p*<0.01) and *(*p*<0.05) indicate significantly different from wild type or Akita alone as determined by ANOVA followed by Tukey's post test.

To gain better insight into the biochemical basis of the kidney pathology and its improvement by fisetin, we examined a number of markers in the kidney that are related to oxidative stress. The thiobarbituric acid reactive substances (TBARS) assay measures the levels of lipid hydroperoxides. Thus, it is both a general indicator of oxidative stress and also a surrogate marker of GSH availability since lipid hydroperoxides are specifically removed by glutathione peroxidase 4, which is absolutely dependent on GSH [23]. Kidney TBARS levels were increased approximately 2-fold in the Akita mice relative to wild type mice and this was prevented by feeding fisetin (Fig. 3A). Fisetin has no effect on TBARS levels in the wild type mice.

Osteopontin (OPN), a multifunctional phosphoglycoprotein and pro-fibrotic adhesion molecule, is upregulated in the kidneys of diabetic mice and humans, possibly by increases in oxidative stress [24], and is a critical mediator of diabetic nephropathy [25]. As shown in Figure 3B, OPN protein expression was upregulated ∼2 fold in the Akita mice. The up-regulation of OPN in the Akita mice was prevented by fisetin treatment. Fisetin had no significant effect on OPN expression in the wild type mice.

As noted above, the pathological features of diabetic kidney include markers of oxidative stress [26] and fisetin has the ability to maintain the level of the cells' major antioxidant, GSH, under conditions of oxidative stress [5]. The rate-limiting step in GSH synthesis is catalyzed by glutamate cysteine ligase (GCL). Figure 3C shows that diabetes decreased the level of GCL and this was restored by fisetin. There was, however, no change in total GSH levels in the kidney of any of the four groups of animals (not shown), suggesting that the maintenance of GSH homeostasis is dependent on GSH availability rather than steady state GSH levels [27].

**Figure 4:**
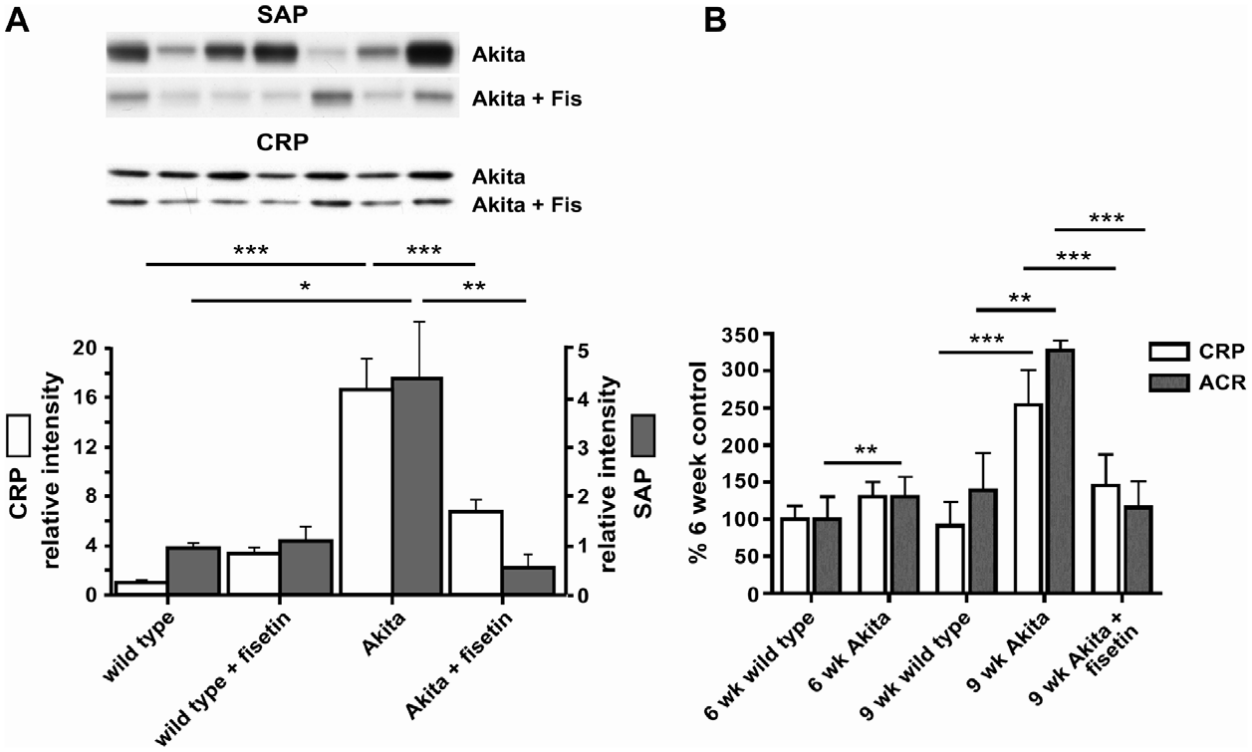
Fisetin reduces markers of inflammation in Akita mice. (A) Male Akita mice were fed control diet or fisetin (0.05%) diet for 18 weeks beginning at 6 weeks of age. C-reactive protein (CRP) and serum amyloid P (SAP) were measured in plasma by Western blotting. Fisetin reduced plasma CRP and SAP levels in the Akita mice to nearly the levels in the control mice. All data are mean ± SD of *n* = 5–7/group. ***(*p*<0.001) indicates significantly different from wild type or Akita alone as determined by ANOVA followed by Tukey's post test. (B) Age dependent increases in plasma CRP and urine albumin/creatinine ratio (ACR). Male Akita mice were first tested at 6 weeks of age and then fed control diet or fisetin (0.05%) for 3 weeks prior to re-testing. C-reactive protein (CRP) was measured in plasma by Western blotting. The albumin/creatinine ratio (ACR) in the urine was determined using ELISAs. All data are mean ± SD of *n* = 8–10/group. **(*p*<0.01 - 6 week) indicates significantly different from wild type as determined by t-test. **(*p*<0.01) and ***(*p*<0.001) indicate significantly different from wild type or Akita alone as determined by ANOVA followed by Tukey's post test.

An additional marker for oxidative stress that has been implicated in the complications of diabetes is the receptor for advanced glycation end products (RAGE). Activation of RAGE is pro-oxidant and pro-inflammatory and is being investigated as a therapeutic target for diabetic complications [28]. Figure 3D shows that RAGE is highly elevated in the Akita mouse kidneys and that fisetin significantly lowers RAGE expression. Since elevated RAGE is also a physiological response to both inflammation and protein glycation, we examined another indicator of inflammation, the acute phase response (APR) protein C-reactive protein (CRP), in the blood of the Akita and control mice. CRP is a blood marker for the chronic systemic inflammation associated with diabetes and elevated serum CRP is also associated with both a decline in cerebral integrity and cognitive function [29]. As shown in Figure 4A, CRP was significantly increased in the plasma of Akita mice and this increase was attenuated by the fisetin-rich diet. In contrast to humans, the major APR protein in mouse blood is serum amyloid P (SAP) rather than CRP [30] although the expression of both proteins is coordinately regulated by the same transcription factor [31]. As shown in Figure 4A, SAP was regulated in a manner very similar to CRP supporting the idea that the increase in CRP reflects a systemic inflammatory response.

To determine the relationship between kidney dysfunction and systemic inflammation as indicated by CRP levels, we compared the albumin/creatinine ratio in the urine of 6 and 9 week old Akita mice to their plasma CRP levels. As shown in Figure 4B, there was a close association between the increase in plasma CRP and albuminuria. Furthermore, while the 9-week old Akita mice showed a significant increase in both plasma CRP and albuminuria, this was largely blocked by the feeding of fisetin beginning at 6 weeks of age.

### Fisetin reduces MG-dependent protein glycation

**Figure 5:**
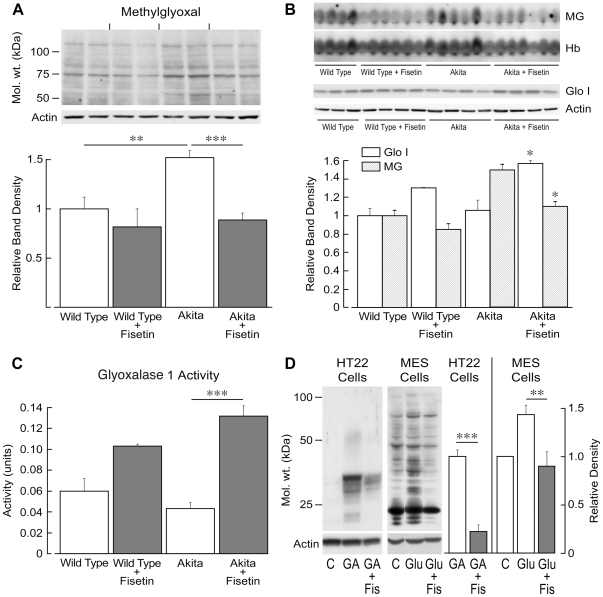
Fisetin reduces MG-dependent protein glycation in the kidney and blood. (A) Kidneys were homogenized in PBS containing phosphatase and protease inhibitors and equal amounts of protein were analyzed by SDS-PAGE and Western blotting with an antibody to MG and actin as a loading control. The entire gel as shown was scanned and the total density normalized to actin. Four to 6 animals were used per group for quantifying band densities with 2 representative kidneys per group shown. (B) Either whole blood was assayed by Western blotting with an anti-MG antibody or kidney tissue with an anti-glyoxylase 1 antibody. The major MG-glycated protein in blood was hemoglobin (shown) and its loading control is total hemoglobin (α and β). Glyoxylase 1 expression was normalized to actin. (C) The activity of glyoxylase 1 was measured and is expressed in units. One unit is the amount of activity required to produce 1 mmole of S-D-lactoylglutathione/min/mg protein. ***(*p*<0.001), **(*p*<0.01) and *(*p*<0.05) indicate significantly different from wild type or Akita alone as determined by ANOVA followed by Tukey's post test. (D) Exponentially dividing HT22 cells were exposed to 5 mM glutamic acid (GA) with or without 2 µM fisetin (Fis) for 8 h. The cells were lysed in SDS sample buffer and equal amounts of protein were analyzed by SDS-PAGE and Western blotting with an antibody to MG and actin as a loading control (n = 3). The kidney mesangial cell line MES13 was grown in 5 mM glucose (glu) (C is control) and some cells transferred to 25 mM glucose plus or minus 10 µM fisetin (Fis) for 8 h. The cells were lysed is SDS sample buffer and equal amounts of protein were analyzed by SDS-PAGE and Western blotting with an antibody to MG and actin as a loading control (n = 3). For both cell lines, the entire gels as shown were scanned and the total densities normalized to actin. Significant differences were determined by ANOVA followed by Tukey's post test. **p<0.01; *** *p*<0.001.

Because RAGE expression is elevated by protein glycation and reduced by fisetin (Fig. 3D), we investigated whether fisetin inhibits MG-dependent protein glycation, a potential causative agent in the complications of diabetes [11]. Proteins glycated by MG were assayed in kidney homogenates and whole blood of Akita mice by Western blotting using a monoclonal antibody against the N-acetyl-N(5-hydro 5-methyl)-4 imidazolone adduct of MG [32]. Figure 5A shows that there is a significant increase in MG-glycated proteins in the kidneys of Akita mice that is reduced to control levels in mice fed fisetin. There is also a significant increase in MG-glycated hemoglobin in Akita mice (Fig. 5B). Fisetin significantly lowers the level of MG-glycated hemoglobin in wild type mice and reduces it in Akita animals (Fig. 5B). There is not, however, an increase in the ratio of MG-glycated to total hemoglobin in the Akita animals because hemoglobin levels are also increased, perhaps as a compensatory mechanism for the protein damaged by glycation. The reduction of MG-glycated hemoglobin by fisetin is, however, physiologically important because glycated proteins increase oxidative stress and inflammation by the activation of RAGE. In contrast, there is no significant change in the HbA1c values in fisetin-treated mice (Table 1). However, the HbA1c assay, which is used as an index of blood glucose levels in diabetic individuals, detects chemically reversible, early glycosylation products rather than irreversible AGEs [33]. In addition, fisetin increases both the amount and activity of Glo-1 in kidney tissue (Fig. 5B and C).

It is possible that the ability of fisetin to lower MG-dependent protein glycation in the kidney and blood is independent of a direct effect on MG-dependent glycation at the cellular level. To rule out this possibility, the ability of fisetin to inhibit MG-dependent protein glycation caused by GSH depletion and by high glucose was examined in cultured cell lines. HT22 is a nerve cell line in which exogenous glutamic acid (GA) inhibits cystine uptake which is required in its reduced form for GSH synthesis. GSH depletion leads to a type of programmed cell death called oxytosis [34] and it also leads to the accumulation of MG-glycated proteins (Fig. 5D). However, in the presence of 2 µM fisetin, MG-dependent protein glycation is greatly reduced. Similarly, high glucose leads to an increased flux of glucose through the glycolytic pathway, increased MG formation and more proteins glycated by MG [10]. When kidney mesangial cells grown in 5 mM glucose are exposed to 25 mM glucose, there is an increase in MG-dependent protein glycation that is reduced by 10 µM fisetin (Fig. 5D). These data show that fisetin has a direct effect at the cellular level on protein glycation by MG that is independent of the diabetic state in animals.

**Figure 6:**
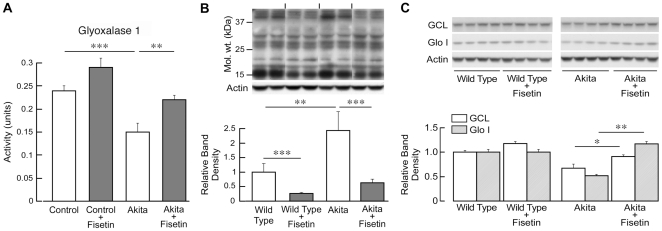
Fisetin reduces MG-dependent protein glycation in the cortex. Brain cortical extracts were prepared and analyzed exactly as for the kidney shown in Fig. 5 with 6 to 8 animals per group. (A) Glyoxylase 1 enzymatic activity. (B) Protein analyzed by SDS-PAGE and Western blotting with an antibody to MG with actin as a loading control. The entire gel as shown was scanned and the total density normalized to actin. (C) Western blotting with anti-GCL and Glo-1 antibodies. In B and C, the data are presented graphically as relative band density of indicated protein over actin. ***(*p*<0.001), **(*p*<0.01) and *(*p*<0.05) indicate significantly different from wild type or Akita alone as determined by ANOVA followed by Tukey's post test.

Figure 2 shows that fisetin has the ability to reduce an experimental index of anxiety, an indicator of CNS dysfunction, in Akita mice. Therefore, it was asked whether fisetin alters the parameters of MG metabolism in the brain as it does in the kidney. Figure 6A shows that the enzymatic activity of Glo-1 is decreased in the cortices of Akita mouse brains and is elevated by fisetin. Consistent with the decrease in Glo-1 activity in the Akita mouse brains, there is a significant increase in MG-glycated proteins in the cortex (Fig. 6B) that is decreased to control levels by fisetin. Furthermore, there is also a highly significant decrease in MG-glycated proteins in the cortex from fisetin-treated wild type mice (Fig. 6B). Finally, both Glo-1 and GCL protein levels are decreased in the cortices of Akita mouse brains relative to those of wild type mice and are restored to control levels by fisetin in the diabetic mice. Together these data show that the regulation of MG metabolism by fisetin is similar in brain and kidney and support the recent observation that kidney disease is a marker for cognitive problems in patients with type 1 diabetes [13].

## Discussion

The major findings of this study are that fisetin can prevent the development of several indices of diabetic complications when orally administered to type 1 diabetic Akita mice and that this therapeutic outcome is associated with a decrease in MG-dependent protein glycation. Since a single molecule is effective against multiple manifestations of two distinct complications of diabetes, these results lend support to the hypothesis that many of the complications of diabetes share common pathogenic mechanisms [3]. Fisetin increases the expression of both the rate-limiting enzyme in GSH synthesis, GCL, and the MG-degrading enzyme, Glo-1, that requires GSH as a co-factor. Since fisetin concomitantly reduces protein glycation by MG, it is likely that MG-dependent protein glycation contributes to both the neurological and kidney complications of diabetes. These results also provide evidence that a single, multi-target compound is a viable approach to the treatment of distinct, tissue-specific diabetic complications.

Although overexpression of Glo-1 has been shown to reduce indices of diabetic complications both *in vitro* [32], [35] and *in vivo* [36], it has not been clear how to translate those results into the clinic. In addition, a number of flavonoids [37] and other natural products such as curcumin [38] have been reported to inhibit Glo-1 activity as has the insulin-sensitizing drug troglitazone [39]. In contrast, not only does fisetin increase Glo-1 levels and activity but it also increases the synthesis of its essential co-factor GSH. Thus, the use of fisetin represents a unique approach to enhancing the function of the glyoxalase pathway.

We employed the Akita model of type 1 diabetes to obviate any concerns about direct organ toxicity arising from the use of diabetogenic chemicals. As reported previously, the Akita mice showed a number of changes consistent with diabetic nephropathy [14], [25]. In our study, these included significant increases in kidney weight, TBARS levels and OPN expression as well as an increase in the urine albumin/creatinine ratio. However, while the effects of diabetes on kidney weight and urine albuminuria were marked, increases in glomerular volume and mesangial score were much less robust, suggesting that these mice exhibit a slowly progressing, mild functional nephropathy. Most of the biochemical and functional consequences of diabetes in the kidney were reduced or prevented by fisetin. The effects of fisetin on OPN expression are consistent with a recent study showing that OPN deletion on the Akita background attenuated the effects of hyperglycemia on multiple aspects of diabetic nephropathy including kidney weight and proteinuria [25] suggesting that this protein may be a key target of the actions of fisetin.

Although less well studied than other complications, the CNS complications of diabetes can also have a significant impact on the quality of life of patients with the disease [13]. Fisetin is unique among the natural products previously tested for their effects on diabetic complications in that it also exhibits neurotrophic and neuroprotective properties in CNS neurons [40], enhances memory [17] and improves behavioral outcomes following ischemic stroke [41]. The neurotrophic activity of fisetin may contribute, along with its ability to reduce MG-dependent protein glycation, to its efficacy against the reduced open field behavior seen in the Akita mice, as efficacy cannot be attributed to improvement of systemic indices of diabetes.

Methylglyoxal is a toxic α-oxoaldehyde glycating agent mostly formed from triose-phosphates and it has been suggested that MG-dependent protein glycation is responsible for the majority of the pathology associated with diabetic complications [11], [35], [12]. It is shown here that glycation of proteins by MG is increased in Akita blood, kidney and brain, and that this increase is reduced by fisetin (Fig. 5A, B; Fig. 6B). Elevated exogenous glucose increases the rate of glycolysis and the greater the flux of glucose through the glycolytic pathway, the greater amount of MG is produced [10]. In addition, if glycolysis is inhibited downstream of the triose-phosphates by the oxidative inactivation of GAPDH, there is a backward flux of trioses and increased MG production. Oxidative stress is elevated in diabetes [3] (Fig. 3A), GAPDH is inactivated under conditions of mild oxidative stress [42], and fisetin has dual antioxidant functions by maintaining intracellular GSH levels and acting as a direct antioxidant [5]. Glo-1 is the rate-limiting enzyme in the removal of MG and it is dependent upon reducing equivalents derived from NADPH through its reduction of oxidized GSH. Fisetin also increased the expression and activity of Glo-1 as well as GCL, the rate-limiting enzyme in GSH synthesis (Fig. 3C, Fig. 5B, C, Fig. 6A, C). Oxidative stress and protein glycation caused by hyperglycemia also induce the expression of the RAGE protein in the kidney, which is greatly reduced by fisetin (Fig. 3D). In summary, these data argue that MG-dependent protein glycation is a major therapeutic target of fisetin.

Fisetin also exhibits a number of related properties that may contribute to its efficacy in addition to the reduction of MG-dependent protein glycation. The increase in plasma CRP and SAP levels seen in our Akita mice and its prevention by fisetin treatment are novel findings, suggesting that the Akita mice are in a systemic pro-inflammatory state that can be prevented by fisetin. This conclusion is supported by the data from the 6 and 9 week old Akita mice which showed an increase in plasma CRP with age which was blocked by fisetin (Fig. 4B). Increases in markers of systemic inflammation are associated with both oxidative stress in the microvasculature and diabetic complications [43]. Protein glycation is a major contributor to inflammation because it activates RAGE, resulting in both oxidative stress via NADPH oxidase and inflammation through the activation of the pro-inflammatory transcription factor nuclear factor kappa B [8]. Furthermore, CRP can directly increase ROS production by endothelial cells [44]. Overexpression of OPN is also associated with chronic inflammation [45], oxidative stress [46] and GSH depletion [46]. An interaction between systemic and tissue specific inflammation is supported by the results showing a coordinated increase in albuminuria and plasma CRP in Akita mice as they age. The effects of fisetin on both plasma levels of CRP and kidney expression of OPN as well as albuminuria suggest that it has both systemic and tissue-specific effects on inflammation that can reduce the consequences of diabetes. These anti-inflammatory effects may also be related to the reduction in anxiety associated behavior seen in the fisetin-treated Akita mice since hypothalamic pituitary-adrenal axis activation, which is implicated in anxiety-associated behavior in the Akita mice [15], is induced under pro-inflammatory conditions [47]. Fisetin therefore has the potential to disrupt both pro-inflammatory and pro-oxidative pathways that are implicated in the complications of diabetes.

Flavonoids are extensively metabolized following oral consumption resulting in glucuronidated, sulfated and methylated metabolites (for reviews see [48], [49]). The metabolism of fisetin was recently characterized in rats following intravenous and oral administration [50]. Following both types of administration, the levels of free fisetin in serum declined rapidly while the levels of sulfated/glucuronidated fisetin increased. Following oral administration at 50 mg/kg, the serum concentration of fisetin sulfates/glucuronides was maintained at ∼10 µM for >24 hr. When tested in an assay for antioxidant activity, the fisetin sulfates/glucuronides showed somewhat reduced but still significant activity as compared to free fisetin [50].

In summary, we show that oral administration of fisetin significantly reduced indices of two of the complications of diabetes. Fisetin also coordinately decreased the level of MG-dependent protein glycation in kidney, brain and blood, suggesting that MG may be a common pathological factor that contributes to multiple complications of diabetes. Therefore, fisetin or a synthetic derivative might be useful for the treatment of diabetic patients. Although fisetin is not particularly abundant in many fruits and vegetables, the incorporation of significant quantities of fisetin-rich foods into the diet of diabetic patients might provide an alternative approach. The highest levels of fisetin (160 µg/g) are found in strawberries with 5–10 fold lower levels in apples and persimmons [51] and smaller amounts in kiwi fruit, peaches, grapes, tomatoes, onions and cucumbers. The bioavailability of fisetin from these sources has yet to be studied but may be a fruitful area of research.

## Materials and Methods

### Mice

C57BL/6-*Ins2^Akita^* (Akita) mice in the C57BL/6 background and C57BL/6 control mice were purchased from The Jackson Laboratories (Bar Harbor, ME). *Ins2^Akita^* is a model of type 1 diabetes [52]. The Akita spontaneous mutation is an autosomal dominant mutation in the insulin II gene. This missense mutation results in an amino acid substitution that corresponds to the seventh amino acid position of the insulin II A chain. Male mice were maintained under a 12-h light/dark cycle and fed a high fat mouse chow (30E, Harlan) without or with fisetin supplementation (0.05%) ad libitum beginning at 6 weeks of age until they were killed at 24 weeks of age. During the course of the study 2/9 Akita and Akita+fisetin mice died from undetermined causes. Based on average weekly food consumption and body weight, the wild type mice received ∼25 mg/kg fisetin/day while the diabetic mice ate more food and received ∼40 mg/kg/day. The animal studies and the protocols were approved by the IACUC of the Salk Institute (protocol #09-025; approved 5/1/2009).

Blood was taken for determination of glucose, hemoglobin glycation and markers of inflammation at 3 months and then just before sacrifice. Beginning 1 week before sacrifice, urine was collected at exactly the same time of day for 5 days and pooled for analysis. In parallel studies, blood and urine samples were obtained from mice at 6 and 9 weeks of age.

### Measurement of urine albumin and creatinine

Urine albumin concentration was determined by competitive enzyme-linked immunoadsorbent assay using an Albuwell M kit (Exocell, Philadelphia, PA). Urine creatinine concentration was determined by Jaffe's reaction of alkaline picrate with creatinine using a Creatinine Companion kit (Exocell). Results are expressed as the urine albumin-to-creatinine ratio (micrograms per milligram).

### Kidney Morphology

The harvested kidneys were fixed in buffered formalin. After paraffin embedding, 3-micrometer sections were stained with PAS. Morphometry of sections of kidneys was carried out as previously reported [53]. Briefly, twenty-five randomly selected glomeruli in the outer cortex of each kidney section were evaluated in a blinded manner. An image of each glomerulus was overlaid with grids. Each grid intersection was determined as one of the following: capillary lumen, periodic acid-Schiff (PAS) positive mesangial area or nucleus. To evaluate PAS positive area, the total number of PAS positive grid intersections inside each glomerulus was counted. The number of all the grid intersections was calculated for glomerular size.

### Kidney and Brain Protein Analysis

Kidneys or brain cortices were homogenized in phosphate buffered saline containing protease and phosphatase inhibitors. Equal amounts of protein, as determined by the BCA assay (Pierce), were solubilized in SDS-sample buffer containing 0.1 mM Na_3_VO_4_ and 1 mM phenylmethylsulfonyl fluoride, boiled for 5 min and either analyzed immediately or stored frozen at −70°C. Proteins were separated on 10% SDS-polyacrylamide gels and transferred to nitrocellulose. Equal loading and transfer of the samples was confirmed by staining the nitrocellulose with Ponceau-S. Transfers were blocked for 1 h at room temperature with 5% nonfat milk in TBS/0.1% Tween 20 and then incubated overnight at 4°C in the primary antibody diluted in 5% BSA in TBS/0.05% Tween 20. The primary antibodies used were: rabbit anti-osteopontin (AB10910; 1/10,000) and mouse anti-RAGE (MAB5328; 1/12,500) from Millipore (Temecula, CA), mouse anti-MG (Dr. Michael Brownlee; 1/1000), rabbit anti-GCL (sc22663; 1/1000), rabbit anti-glyoxalase-1 (sc67351; 1/1000), goat anti-hemoglobin a (sc31332, 1/1000) and goat anti-hemoglobin b (sc31116, 1/1000) from Santa Cruz (Santa Cruz, CA) and HRP-conjugated rabbit anti-actin (#5125, 1/20,000) from Cell Signaling (Danvers, MA). The transfers were rinsed with TBS/0.05% Tween 20 and incubated for 1 h at room temperature in horseradish peroxidase-goat anti-rabbit or goat anti-mouse (Biorad, Hercules, CA) diluted 1/5000 in 5% nonfat milk in TBS/0.1% Tween 20. The immunoblots were developed with the Super Signal reagent (Pierce, Rockford, IL).

### TBARS Assay

Thiobarbituric acid reactive substances (TBARS), a measure of lipid peroxidation, was assayed in kidney extracts using a kit (Cayman Chemicals, Ann Arbor, MI) and by following the manufacturer's instructions.

### Measurement of C-reactive protein and serum amyloid P

C-reactive protein and serum amyloid P levels were determined by SDS-PAGE and Western blotting analysis of mouse plasma. The primary antibodies used were goat anti-CRP (AF1829; 0.25 µg/ml) from R&D (Minneapolis, MN) and rabbit anti-SAP (A00701; 1/1000) from GenScript (Piscataway, NJ).

### Open field test

The open field test was performed using MED Associates hardware and the Activity Monitor software according to the manufacturer's instructions (MED Associates Inc, St. Albans, VT, USA). The open-field activity monitoring system contains a subject containment environment (chamber), infrared (I/R) sources and sensors. To perform the test, each mouse was placed in the testing chamber and the activity tracked by the Activity Monitor software for 30 min. The scanning was done every 50 ms.

### Cell culture

The kidney mesangial cell line SV40 MES13 (ATCC #CRL-1927) was grown as described in DME containing 5 mM glucose [54]. The cells were then transferred to the same medium containing 30 mM glucose and 8 h later the cells lysed in SDS sample buffer and run on 12% polyacrylamide gels for Western blotting using the anti-MG monoclonal antibody [32]. HT22 mouse hippocampal neurons were grown in DME plus 10% fetal serum [55]. To deplete GSH, cells were exposed to 5 mM glutamic acid and lysed in sample buffer as described above for Western blotting with the anti-MG antibody.

### Glyoxalase-1 assay

The glyoxalase-1 assay was performed using a spectrophotometric method monitoring the increase in absorbance at 240 nm due to the formation of *S*-D-lactoylglutathione for 2 min at 25°C. The standard assay mixture contained 8 mM MG, 2 mM glutathione, 10 mM magnesium sulfate and 50 mM phosphate potassium, pH 6.6. Before initiating the reaction by adding the kidney or brain extract (10–30 µg) to the assay mixture, the mixture was allowed to stand for at least 2 min to ensure the equilibration of hemithioacetal formation. One unit of activity is defined as the formation of 1 mmol of *S*-D-lactoylglutathione/min/mg cell protein. Protein concentration was determined by the BCA assay.

### Statistical Analysis

All groups were compared against each other using one-way ANOVA followed by Tukey's or Dunnett's post-hoc test. Data are presented as group mean ±1 standard deviation.
